# Neuronal Receptors Display Cytoskeleton-Independent Directed Motion on the Plasma Membrane

**DOI:** 10.1016/j.isci.2018.12.001

**Published:** 2018-12-05

**Authors:** Ruth D. Taylor, Martin Heine, Nigel J. Emptage, Laura C. Andreae

**Affiliations:** 1 Centre for Developmental Neurobiology, Institute of Psychiatry, Psychology and Neuroscience, King's College London, London SE1 1UL, UK; 2 MRC Centre for Neurodevelopmental Disorders, King's College London, New Hunt's House, Guy's Campus, London SE1 1UL, UK; 3 Leibniz Institute of Neurobiology, Research Group Molecular Physiology, Brenneckestrasse 6, Magdeburg 39118, Germany; 4 Otto von Guericke University Magdeburg, Center for Behavioral Brain Sciences (CBBS), Universitätsplatz 2, Magdeburg 39106, Germany; 5 Johannes Gutenberg University Mainz, Institute for Developmental Biology and Neurobiology, AG Funktional Neurobiology, Hanns-Dieter-Hüsch Weg 15, Mainz 55128, Germany; 6 Department of Pharmacology, University of Oxford, Mansfield Road, Oxford OX1 3QT, UK

**Keywords:** Neuroscience, Molecular Neuroscience, Optical Materials

## Abstract

Directed transport of transmembrane proteins is generally believed to occur via intracellular transport vesicles. However, using single-particle tracking in rat hippocampal neurons with a pH-sensitive quantum dot probe that specifically reports surface movement of receptors, we have identified a subpopulation of neuronal EphB2 receptors that exhibit directed motion between synapses within the plasma membrane itself. This receptor movement occurs independently of the cytoskeleton but is dependent on cholesterol and is regulated by neuronal activity.

## Highlights

• pH-sensitive quantum dot allows surface-specific single-particle tracking
• EphB2 receptors undergo directed motion on the surface of neuronal membranes
• Directed motion is dependent on membrane cholesterol but not the cytoskeleton
• Directed motion occurs on dendrites between synapses and is activity dependent

## Graphical Abstract

**Figure:**
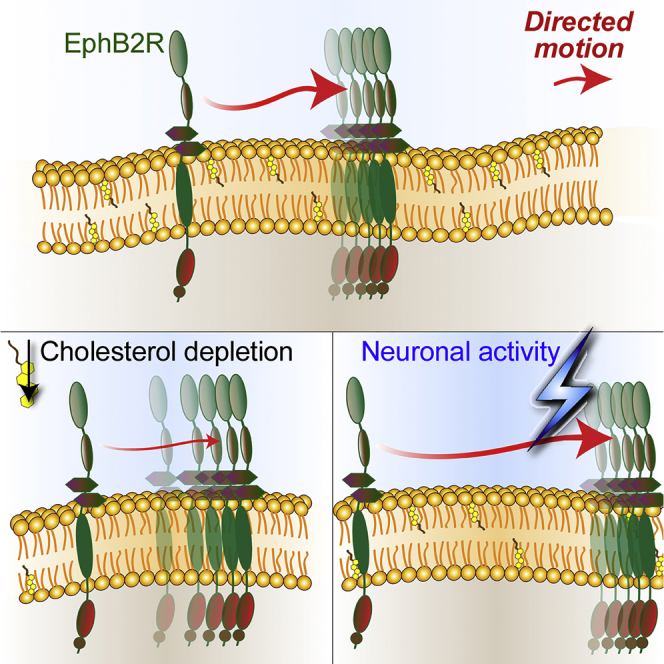

## Introduction

Receptor proteins that sit on the surface of the plasma membrane are critical for enabling cellular responses to the external environment. All cells need to be able to actively move these receptors from one location to another, often in a highly dynamic way. This directed movement is believed to be carried out by an elaborate system involving internalization of receptors by endocytosis into specialized transport vesicles, travel along the cytoskeletal network of the cell driven by molecular motors (Hirokawa and Takemura, 2005, Kapitein et al., 2010), and subsequent re-exocytosis into the destination location in the membrane. This transport is especially important in highly polarized cells such as neurons, where, for example, the rapid delivery of key receptors into and out of synapses can be critical for neuronal function. Indeed, the first description of a molecular motor that transports organelles along microtubules, kinesin, was in neuronal axons, where it mediated anterograde transport from the cell soma to neuron terminals, toward the plus end of microtubules (Vale et al., 1985).

In addition, membrane proteins exhibit lateral diffusion within the plasma membrane (Borgdorff and Choquet, 2002, Dahan et al., 2003, Heine et al., 2008, Kusumi et al., 1993). Lateral diffusion refers to the passive, random movement of proteins on the plasma membrane where they can move freely unless hindered sterically. This diffusional behavior was demonstrated using predominantly two main approaches: fluorescence recovery after photobleaching (FRAP) (Goehring et al., 2010) and single-particle tracking (SPT) (Triller and Choquet, 2008). FRAP experiments require the ensemble averaging of multiple molecules, whereas SPT allows tracking of individual proteins with a single fluorophore. The development of brightly fluorescent semiconductor nanoparticles, or quantum dots (QDs), with exceptional photostability and narrow emission spectra allowed accurate fluorophore localization (<10 nm) (Suzuki, 2012), long recording periods (>10 min), and improved color multiplexing in SPT. In neurons, the lateral diffusion of receptors can be restricted by membrane structures such as synapses and lipid rafts (Triller and Choquet, 2008). Furthermore, surface diffusion of these receptors and their recruitment into synapses are known to play important roles in synaptic plasticity and learning behavior (Choquet and Triller, 2013, Penn et al., 2017).

Overall, these findings have led to a current model where transmembrane proteins are believed to diffuse randomly in the plasma membrane but are transported in a directed manner within the cell via the movement of transport vesicles. However, it is not clear whether receptors might be able to undergo directed movement on the surface of the membrane. There is limited evidence that GABA_A_ receptors may show this kind of behavior in neuronal growth cones (Bouzigues et al., 2007), but this was clearly entirely dependent on the microtubule network. In addition, given that QD-based probes are also able to report individual receptor switching between surface diffusion and intracellular active motor transport (Vermehren-Schmaedick et al., 2014), a confident description of movement on the membrane surface requires surface-specific protein tracking. We now describe a specific pH-sensitive QD probe that can distinguish between surface membrane dynamics and intracellular transport. Using this probe, we adopted an SPT approach to track the transmembrane tyrosine kinase receptor, EphB2, a well-known neuronal protein with important roles in synapse formation (Sheffler-Collins and Dalva, 2012) and plasticity (Contractor et al., 2002, Grunwald et al., 2001), whose active intracellular transport along the cytoskeleton is important for dendritic arbor formation in neurons (Hoogenraad et al., 2005). We applied a sliding window analysis method to analyze the membrane surface trajectories, revealing directed motion of EphB2 receptors (EphB2Rs) on the cell surface of neuronal dendrites, occurring almost exclusively between synapses. This surface-directed motion was modulated by the cholesterol composition of the cell membrane but was insensitive to disruption of the cytoskeleton. To our knowledge, this is the first description of transmembrane protein movement on the cell surface by directed motion that is cell cytoskeleton independent. As both the lateral diffusion and intracellular vesicular transport of receptors in neurons are critical for synaptic and neuronal function, and hence regulated by activity (Anggono and Huganir, 2012, Bourke et al., 2018, Choquet and Triller, 2013, Park et al., 2004, Rumpel et al., 2005), we investigated the impact of activity on surface-directed motion. We found that neuronal activity promoted surface-directed movement of EphB2Rs.

## Results

### Surface-Specific Tracking with a pH-Sensitive Quantum Dot

**Figure 1:**
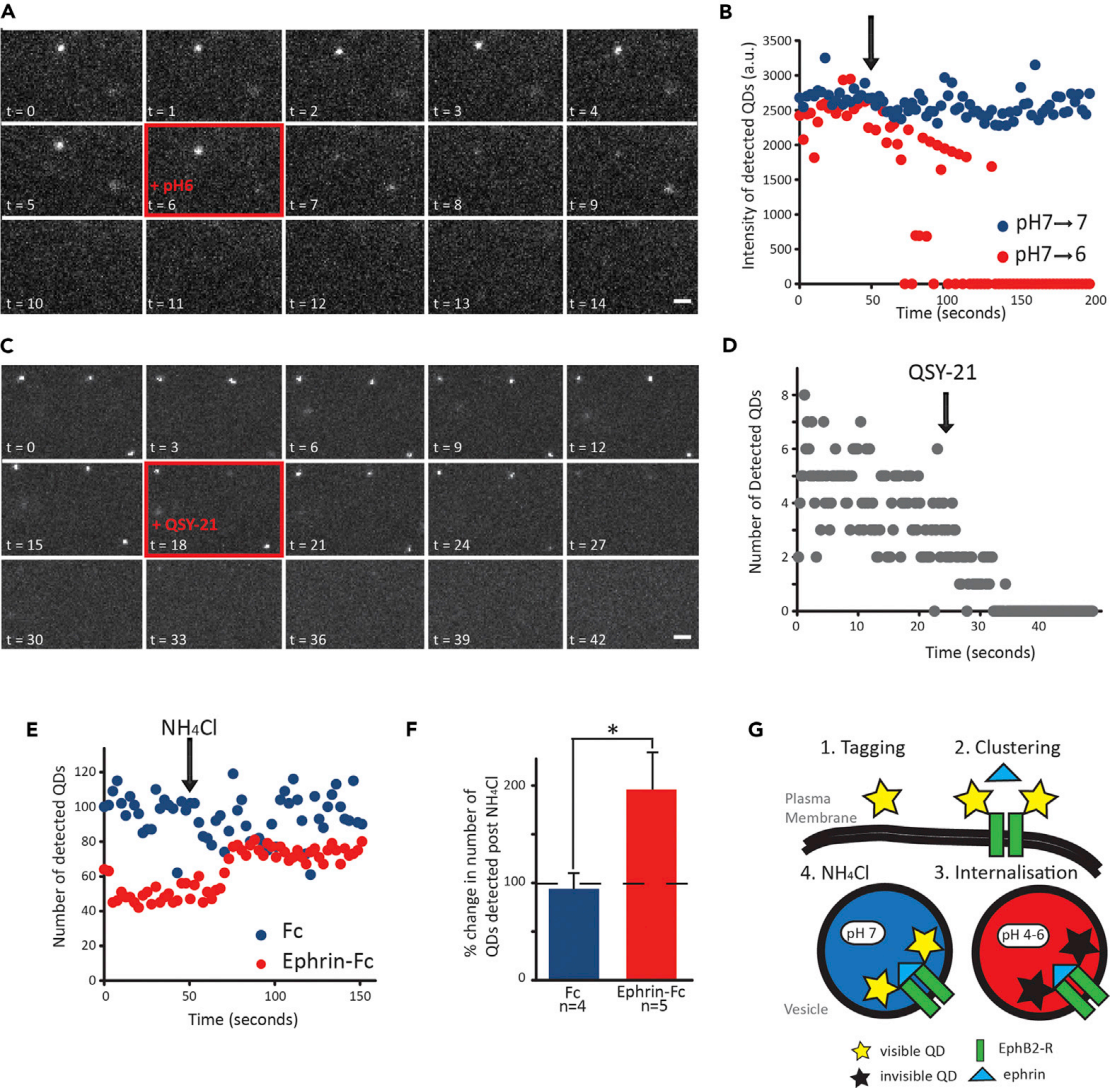
Single-Particle Tracking of EphB2Rs in Hippocampal Neurons with a Surface-Specific Quantum Dot Probe (A and B) (A) Example time-lapse image series (in seconds) and (B) mean fluorescence intensities of QD-EphB2Rs following addition of pH 6 solution (red box in A, red circles in B) compared with pH 7 control (blue circles, B); scale bars, 2 μm. (C and D) (C) Example time-lapse image series (in seconds) and (D) number of detected QD-EphB2Rs following addition of membrane-impermeable QSY-21 (100 μM) (red box in C). (E and F) Dissipation of intracellular pH gradients by application of NH_4_Cl (50 mM, pH 7.2) to neurons previously incubated with pre-clustered ephrinB1 (Ephrin-Fc; red circles, n = 5) or control Fc fragments (Fc; blue circles, n = 4) leads to a significant increase in detected QD-EphB2Rs in the former; (E) mean number of detected QD-EphB2Rs over time; and (F) quantification of change from baseline (p < 0.05, graph shows mean ± SEM). (G) Model illustrating experiments shown in (E and F): clustering of QD-labeled EphB2Rs leads to receptor internalization and hence QD fluorescence quenching. These QD-EphB2Rs are then revealed by neutralization of pH.

To specifically target single-molecule imaging to the cell surface, we were able to take advantage of a probe to differentiate between receptor movement on the neuronal cell surface and that within endosomal transport vesicles. The intraluminal (internal) environment of intracellular vesicles is relatively acidic (pH < 6) (Ouyang et al., 2013). We exploited our observation that the level of fluorescence emitted by the 655-nm-wavelength QD exhibited significant pH sensitivity. We labeled endogenous EphB2Rs in cultured neurons with the QD655 conjugate and examined the effect of changing the external pH. Bath application of pH 6 solution resulted in a dramatic quenching of QD fluorescence intensity to a level at which QDs could no longer be detected, whereas pH 7 solution had no effect (Figures 1A and 1B). As QD655 fluorescence is virtually undetectable at pH 6, this suggested that any visible QDs should reside on the neuronal surface, as those lying within vesicles would be effectively invisible. To verify that this was indeed the case, we used the membrane-impermeable dye, QSY-21, which is known to quench QD fluorescence (Jablonski et al., 2010). Addition of QSY-21 resulted in the inability to detect any QD-EphB2Rs within 10 s of its application (Figures 1C and 1D), further indicating that all visible QD-EphB2Rs are on the cell surface.

To confirm that intravesicular QD-labeled EphB2Rs are not detectable and to assess reversibility of quenching, we pre-treated neurons with clustered ephrinB1-Fc, a high-affinity ligand for EphB2Rs, which has been shown to cluster EphB2Rs and cause internalization to endosomes (Zimmer et al., 2003). If internal QD-EphB2Rs are present, but rendered undetectable by the relatively acidic endosomal environment, we reasoned that they should be revealed by neutralizing the endosomal pH gradient with ammonium chloride (NH_4_Cl) (Axelsson et al., 2001, Miesenbock et al., 1998, Sankaranarayanan and Ryan, 2000). Upon addition of NH_4_Cl, we saw a significant increase in the number of detected QD-EphB2Rs, compared with neurons that were treated with control Fc only (Figures 1E–1G). This unveiling of internalized QD-EphB2Rs further confirms that QD-EphB2Rs traveling within vesicles inside the cell are not visible and indicates that the pH-dependent quenching of QD655 is reversible. Taken together, these experiments demonstrate that all visible QD-EphB2Rs are located on the cell membrane surface.

### EphB2Rs Show Directed Motion on the Surface of Neurons

**Figure 2:**
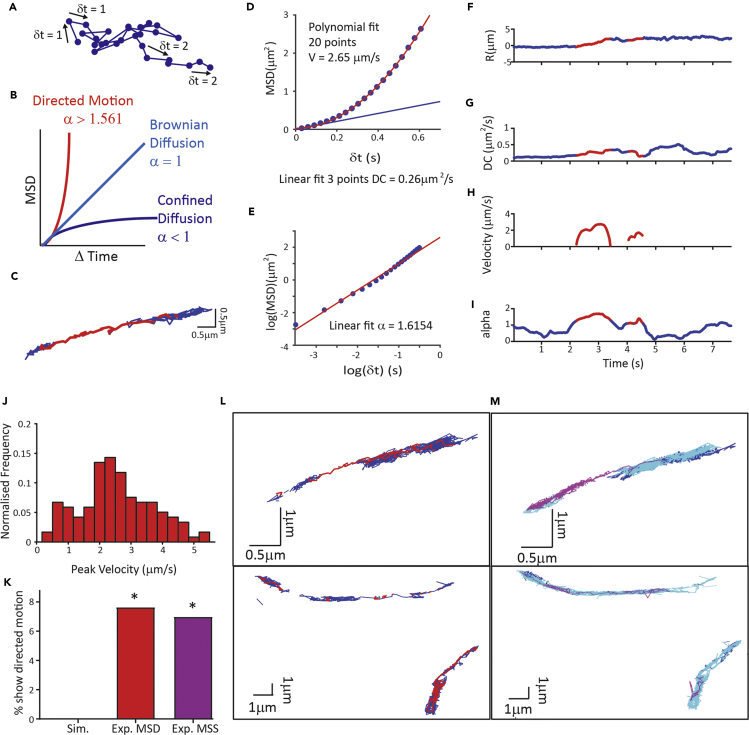
Analysis of QD-EphB2R Trajectories Reveals Directed Motion (A) Model trajectory indicating δt. (B) Traditional analysis plots MSD against Δt to obtain exponent, α, indicating motion type. (C) Example QD-EphB2R trajectory. Colors represent motion modes with red indicating directed motion and blue illustrating diffusive motion. See also Video S1. (D–I) Time resolved MSD analysis. (D) MSD plotted against time interval (δt) for illustrated trajectory during directed motion for a single “sliding” window (20 time points) yields a DC of 0.26 μm^2^/s by linear fit over the linear portion of the graph (blue line) and a velocity (V) of 2.65 μm/s when fitting a power equation to the full dataset (red curve). (E) A plot of log(MSD) against log(δt) for the same period yields alpha (α) of 1.6154 (slope of linear fit). (F–I) Time-resolved MSD analysis of the illustrated QD-EphB2R trajectory seen in (C) showing (F) displacement, (G) diffusion coefficient, (H) velocity, and (I) α varying over the time course of the trajectory; threshold for directed motion α > 1.561. (J) Frequency distribution of detected peak velocities in experimental data (n = 119; mean = 2.51 ± 1.16 μm/s). (K) Quantification to compare percentage of experimental QDs showing directed motion with simulated data of Brownian motion (0/350), time-resolved MSD (red, 14/183, chi-square p < 0.0001), and DC-MSS (magenta, 34/452, chi-square p < 0.0001). See also Figure S1. (L and M) Example trajectories of QD-EphB2Rs displaying similar segments of directed motion using (L) time-resolved MSD analysis and (M) DC-MSS analysis. Trajectories are color coded for motion mode depending on analysis: (L) for time-resolved MSD, directed motion is displayed in red and diffusion in blue, whereas (M) for DC-MSS, directed motion is in magenta, with diffusion in blue and confined diffusion in cyan.

SPT of surface QD-EphB2Rs in hippocampal neurons revealed that individual receptors are not static but move laterally in the neuronal cell membrane (Video S1 and Figure 2C). Standard approaches to the analysis of single-particle movement have utilized the mean squared displacement (MSD) method to distinguish between three types of motion displayed by particles: confined diffusion, free Brownian diffusion, and super-diffusive or “directed” motion. The method plots the MSD against the change in time between each “step” (δt) of the trajectory (Figure 2A) and determines two coefficients that describe the change in MSD. The diffusion coefficient (DC) is calculated over the linear portion (i.e., the first few points) of the function. The exponent α describes the curvature of the function and thus is an indicator of the type of motion displayed by the receptor, where α = 1 represents diffusion, α < 1 indicates confined diffusion, and α > 1 indicates directed motion, and it is resolved over a longer timescale (Saxton and Jacobson, 1997) (Figure 2B). This type of analysis is generally applied over an entire trajectory and therefore assigns a single motion mode to each particle trajectory. However, we noticed that whereas some QD-EphB2Rs were either stationary or mobile for the duration of the recording, others interchanged between periods of relative localized stability and unrestricted motion (Video S1), suggesting that a more time-resolved analysis would be able to better describe individual trajectories. Indeed, segmenting trajectories by synaptic location has previously demonstrated that the diffusion characteristics of neurotransmitter receptors vary depending on whether they are located at or outside the synapse (Renner et al., 2012). We therefore analyzed the trajectories of QD-EphB2Rs by calculating MSD within sliding time windows, based on an approach previously validated in microtubule transport (Arcizet et al., 2008). This approach generates a (smoothed) plot of how each parameter (distance, DC, α) changes with time (Figures 2F–2I) and confirmed our impression that individual receptors exhibit varying DCs over the time course of a single trajectory (Figure 2G).

Video S1. Quantum-Dot-Labeled EphB2Rs Moving on Hippocampal Neurons, Related to Figure 2 Time-lapse imaging of QD-EphB2Rs (red dots) superimposed on bright-field image of dissociated hippocampal neurons. White arrow indicates stationary QD-EphB2R; red arrow indicates QD-EphB2R that displays different patterns of movement during the course of imaging.

To distinguish between diffusive and directed motion, we set a very stringent threshold to define directed motion: as α > 1.56 (see Methods). Nonetheless, to our surprise, we found that some receptors appeared to undergo short periods of directed motion (71/1,287) (Figures 2C–2E and 2I) with an average peak velocity of 2.51 ± 1.156 μm/s (Figure 2J). We compared experimental data (without blinks, see Methods) with time-resolved analysis of simulated trajectories of randomly moving particles displaying Brownian motion. We found that there was a significantly greater proportion of experimental EphB2R trajectories exhibiting periods of directed motion than of simulated ones, indeed with no such episodes arising from analysis of the simulated pure diffusional trajectories (experimental trajectories: 7.7%, 14/183; simulated Brownian trajectories: 0/350, chi-square test, p < 0.0001, Figure 2K). As further confirmation that these surface receptors exhibit directed motion, we employed a second validated approach to analyze QD-EphB2R trajectory data: divide-and-conquer motion scaling spectrum analysis (DC-MSS) (Vega et al., 2018). In summary, DC-MSS initially classifies the motion type by calculating the change in the maximum pairwise distance (MPD) between particle positions within a sliding window. Maxima in ΔMPD indicate a switch of motion modes enabling track segmentation. Once the trajectory has been segmented, the motion scaling spectrum is calculated for each segment of the trajectory as previously described (Ewers et al., 2005). Segments of trajectory that display directed motion analyzed by time-resolved MSD analysis (Figure 2L) similarly display directed motion in DC-MSS (Figure 2M), with a similar proportion of trajectories showing directed motion (7.5%, 34/452, Figure 2K).

To determine whether it might be possible to detect apparent directed motion as a result of the geometry of membrane space available for diffusion, we modeled diffusional movement of particles in a variety of spatially defined regions. In no case were we able to identify directed movement (see Figures S1A and S1B). Reduced sampling rates also only reduced the apparent α (Figure S1C). Since all labeled EphB2Rs in our experiments are restricted to the external neuronal surface, such that the episodes of directed motion cannot be due to intracellular vesicular transport, these results demonstrate that these receptors have the capacity to move with directed motion on the plasma membrane.

### Diffusion of Surface EphB2Rs Is Restricted by Actin, whereas Directed Motion Is Cytoskeleton Independent but Depends on Membrane Cholesterol

**Figure 3:**
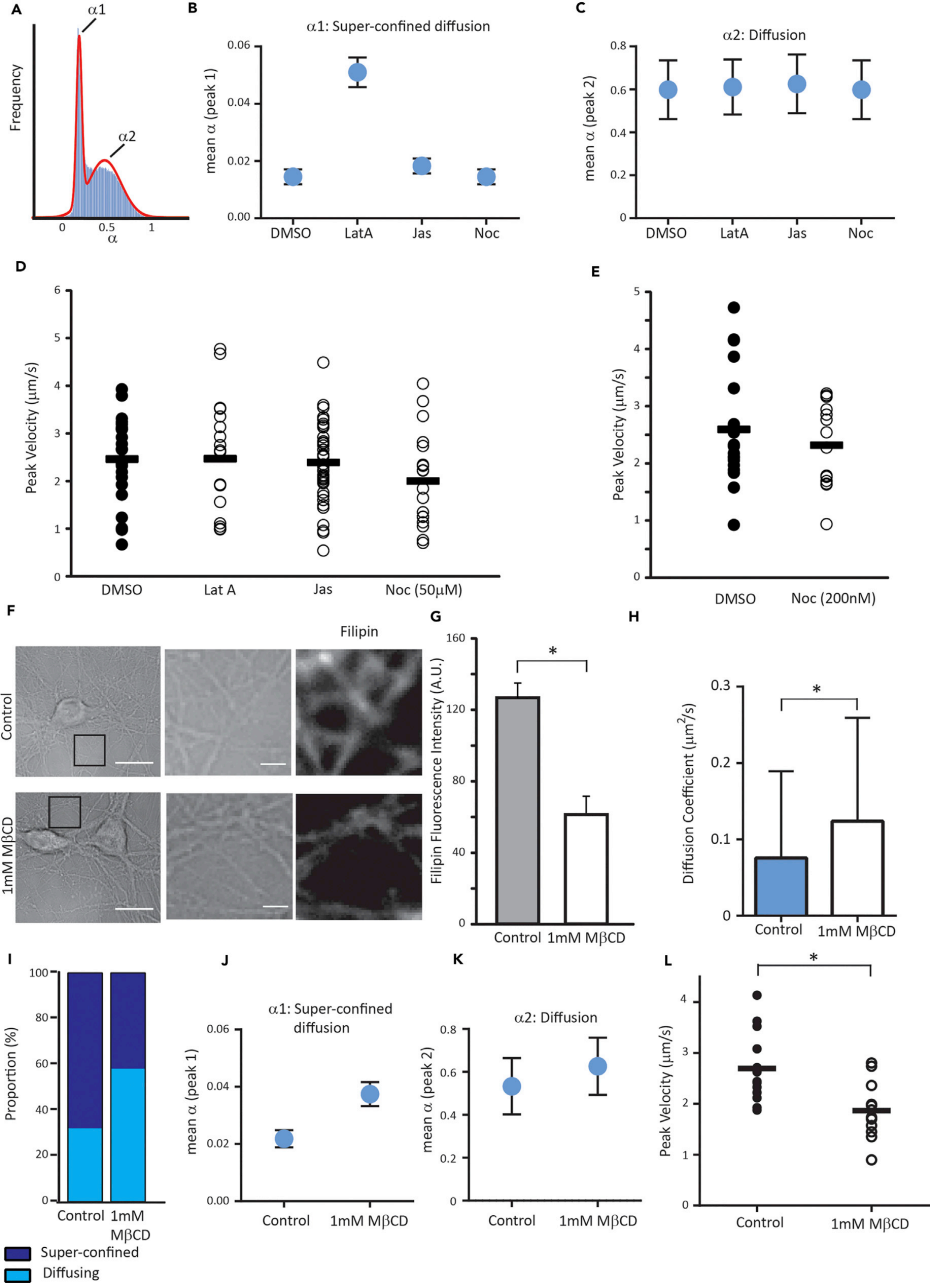
Directed Motion of Surface EphB2Rs Is Not Affected by Cytoskeleton-Modifying Drugs but Is Dependent on Membrane Cholesterol (A) Bimodal frequency distribution plot for values of instantaneous α in diffusing QD-EphB2Rs indicates a super-confined population (α_1_) and a more freely diffusing population (α_2_). (B and C) The effect of modulating the actin cytoskeleton (Lat A: latrunculin A; 5 μM; Jas, jasplakinolide, 5 μM) or the microtubule network (Noc, nocodazole, 50 μM) on α_1_ (B) and α_2_ (C). Error bars represent standard deviation. (D) Modifying the actin cytoskeleton or the microtubule network does not change the peak velocity of QD-EphB2Rs; bars indicate the mean; n = 18–40. (E) As for (D), cells incubated in low-dose nocodazole. (F and G) Treatment with 1 mM methyl-β-cyclodextrin (MβCD) reduces cholesterol levels; (F) left panels show representative bright-field images of dissociated neurons; scale bar, 10 μm, with higher-power views of bright-field images (middle) and filipin fluorescence (right) of selected regions; scale bar 2 μm. (G) Quantification of filipin fluorescence intensities; error bars represent SEM (control: n = 48 dendrites, MβCD: n = 49, p < 0.05). (H–K) (H) Cholesterol depletion with MβCD significantly increases the instantaneous DC of diffusing QD-EphB2Rs (control: 0.075 μm^2^/s, MβCD: 0.123 μm^2^/s, p < 0.05). (I) MβCD treatment changes the relative proportions of diffusing QD-EphB2Rs that display super-confined versus freely diffusing characteristics (as defined by the value of instantaneous α), increases mean α_1_ (J) and to a lesser degree, α_2_ (K). (H–K) Error bars represent standard deviation. (L) Peak velocity of QD-EphB2Rs is significantly reduced by MβCD (n = 13–17, p < 0.05, chi-square). See also Figure S2.

We next asked to what extent either the diffusive or the directed motion of surface EphB2Rs is dependent on the cell cytoskeleton. We conducted QD-EphB2R tracking experiments in the presence of cytoskeleton-modifying drugs and compared their motion characteristics with vehicle control (DMSO). When we analyzed only those receptors undergoing diffusive motion (excluding all directed motion), we found that there were two populations of diffusing QD-EphB2Rs: super-confined (mean α = 0.026) and diffusing (mean α = 0.59) (Figure 3A). Recently, an extension of the fluid mosaic model describing cell membrane structure (Singer and Nicolson, 1972) has been proposed, which describes different compartments within a phospholipid-cholesterol sheet, supported by a meshwork of microtubules and actin filaments (Kusumi et al., 2011). This model envisages membrane structure as being composed of an actin-based “fence” and transmembrane protein “pickets.” We speculated that movement of the super-confined group of receptors might be limited by this actin fence, and indeed, disruption of the actin cytoskeleton by addition of latrunculin A (5 μM) caused a significant increase in α (i.e., greater movement) of the super-confined group (Figure 3B) without affecting the more freely diffusing receptors (Figure 3C). No changes to α were seen following disruption of the microtubule network (Figures 3B and 3C). Similarly, latrunculin A caused a significant increase in the mean DC for both super-confined and diffusing receptors with an equivalent shift in the DC distribution (Figure S2). This is consistent with previous studies (Umemura et al., 2008), including of other neuronal receptors (Rust et al., 2010). However, neither modulation of the actin cytoskeleton (depolymerization with latrunculin A or stabilization with jasplakinolide) nor the microtubule network (depolymerization with high-dose nocodazole or inhibition of microtubule dynamics with nocodazole at low dose; Jaworski et al., 2009) altered the directed motion characteristics of QD-EphB2Rs in terms of the peak velocity (Figures 3D and 3E), the proportion of QDs that show directed motion (DMSO: 8.57%, n = 315; jasplakinolide: 10.20%, n = 245; latrunculin A: 8.28%, n = 145; nocodazole: 7.76%, n = 245), or the time any given QD spent in directed motion mode (DMSO: 0.10 ± 0.01 s, n = 25; jasplakinolide: 0.11 ± 0.01 s, n = 40; latrunculin A: 0.14 ± 0.03 s, n = 18; nocodazole: 0.14 ± 0.03 s, n = 18).

We therefore hypothesized that the directed motion of EphB2Rs might be dependent in some way on the neuronal plasma membrane itself. Application of 1 mM methyl-β-cyclodextrin (MβCD) resulted in depletion of membrane cholesterol in neuronal processes by approximately 50%, as quantified by levels of the macrolide filipin, a cholesterol-binding fluorescent marker (Hering et al., 2003, Maxfield and Wustner, 2012, Renner et al., 2009) (Figures 3F and 3G). As previously reported (Renner et al., 2009), cholesterol depletion with MβCD resulted in an increase in DC that was accompanied by a change in the distribution of α (Figures 3H–3K). Although cholesterol depletion did not affect the proportion of QD-EphB2Rs displaying directed motion, it resulted in a significant reduction in the peak rate of directed motion (Figure 3L). This indicates that the directed movement of surface EphB2Rs is dependent on membrane cholesterol.

### Directed Motion of EphB2Rs Occurs between Synapses and Is Activity Dependent

**Figure 4:**
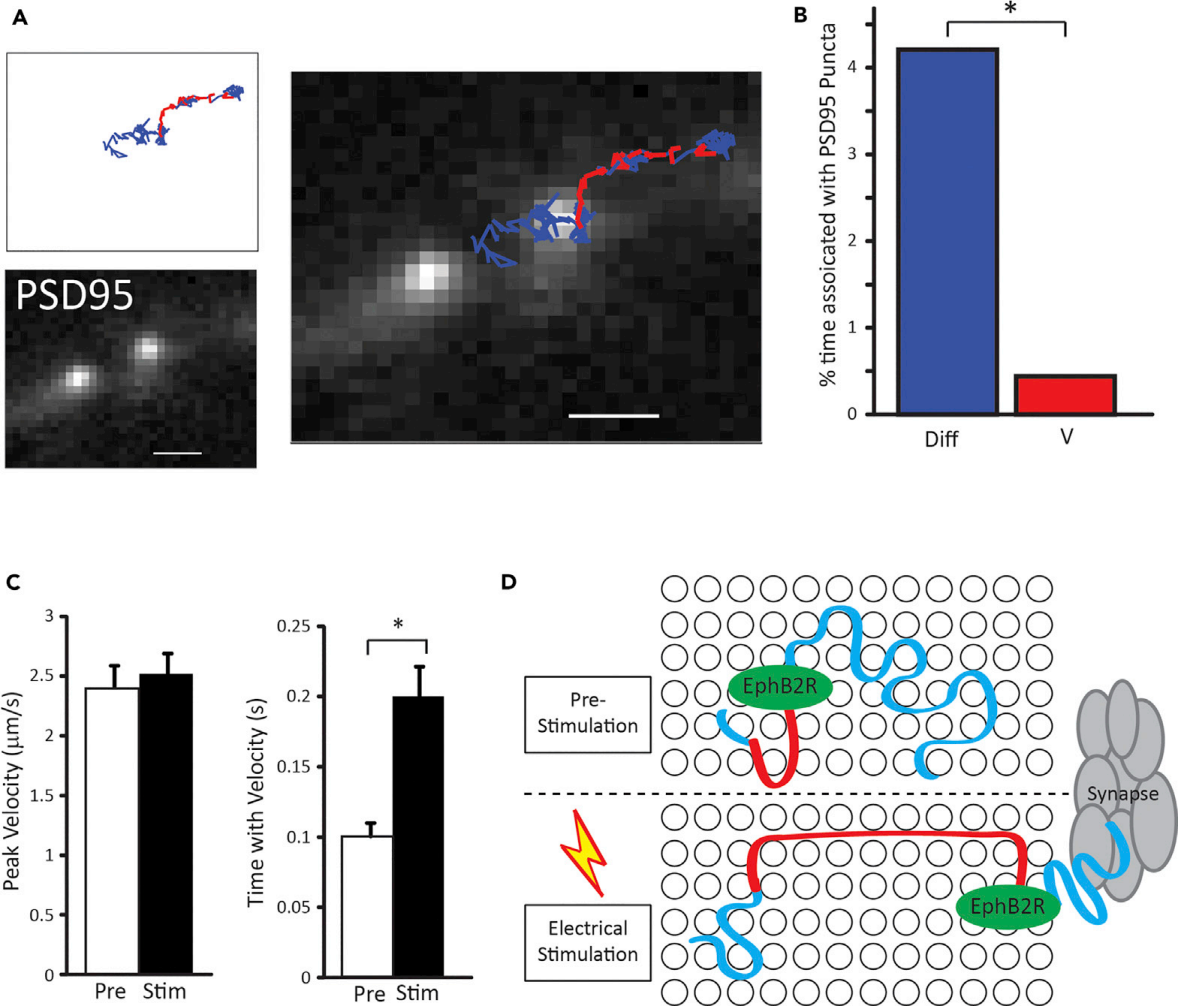
Directed Motion Is Spatially Restricted and Activity Dependent (A) Representative trajectory of a single QD-EphB2R along a neuronal process expressing PSD-95-GFP to localize synapses (see also Figure S3). Regions of the trajectory in which the QD-EphB2R has an associated velocity/displays directed movement are illustrated in red, with diffusive movement in blue; scale bars, 1 μm. (B) Percentage of time in which diffusing QD-EphB2Rs (Diff, dark blue) or those displaying directed motion (V, red) are associated with the peak of PSD-95-GFP puncta (n = 41, p < 0.05, chi-square). (C) Field stimulation of the neuronal network increases time spent with a velocity, without changing the peak velocity. (D) Proposed model for the movement of QD-EphB2Rs in the membrane before and after electrical stimulation. QD-EphB2Rs (shown in green) diffuse in the cell membrane (blue tracks) displaying directed motion (red tracks) for short periods of time. QD-EphB2Rs located near or at the synapse only display diffusive motion. After electrical stimulation, the QD-EphB2Rs spend a greater proportion of time undergoing directed motion. Error bars represent SEM.

We then questioned whether the directed motion of EphB2Rs exhibited any spatial specificity. A key feature of the neuronal landscape is the presence of postsynaptic specializations at intervals along dendrites, which are known to affect receptor diffusion. For example, it has previously been shown that glutamatergic receptors show restricted diffusion within synapses and anomalous diffusion outside synapses (Groc et al., 2004, Heine et al., 2008). Using traditional immunostaining, we find EphB2Rs to be predominantly localized to dendrites, largely between synapses, in these cultures (Figure S3). To track QD-EphB2R movement relative to synapse position, we expressed PSD95-GFP in neurons, to label the postsynaptic compartment, and imaged QD-EphB2Rs. Figure 4A shows a representative trajectory of a QD-EphB2R in relation to PSD-95. In this example, whereas the QD-EphB2R clearly diffuses into the synaptic/perisynaptic compartment (blue), directed motion (red) is only seen outside this region. Quantification of trajectory segments showing diffusion versus directed motion demonstrates that EphB2Rs with a velocity are very rarely seen at the synapse (comprising synapse and perisynaptic area), compared with diffusing receptors (Figure 4B). These data indicate that surface EphB2Rs exhibit fast directed motion along dendrites and between postsynaptic specializations.

Finally, to determine whether this directed movement might be regulated by neuronal activity, we analyzed the directed motion characteristics of QD-EphB2Rs following electrical field stimulation. We found that QD-EphB2Rs travel for a longer duration in directed motion mode following stimulation with 900 action potentials at 20 Hz (mean duration prestimulation: 0.09 ± 0.05 s, mean duration 5 min post stimulation: 0.14 ± 0.09 s) without an associated change in peak velocity (peak velocity prestimulation: 2.40 ± 0.90 μm/s, peak velocity 5 min post stimulation: 2.52 ± 0.73 μm/s) (Figure 4C). Thus increased neuronal activity promotes rapid intersynaptic movement of surface EphB2Rs.

## Discussion

We propose that cholesterol-dependent “conduits” within the dendritic plasma membrane may allow brief spells of rapid surface receptor travel between synapses. Under conditions of increased neuronal activity, receptors spend longer in these conduits (model illustrated in Figure 4D).

In this study, we have used a pH-dependent QD probe to ensure that we have at all times tracked receptors localized to the neuronal plasma membrane. QDs have been described to show pH-dependent changes in fluorescent levels, including quenching at acidic pH (Debruyne et al., 2015, Gao et al., 2002), and have been used previously to examine synaptic vesicle recycling in neurons (Zhang et al., 2009). In the latter study, the QD605 (Fisher) was found to exhibit relatively small levels of quenching (∼15%) at pH 5, although this was still sufficient to detect vesicle cycling. Here, we find that the QD655 exhibits much higher levels of pH sensitivity in this range, making it useful for a variety of biological applications. QD quenching in response to shifts in pH is known to be highly dependent on the nature of the organic coating surrounding the core-shell region, and although the specific coatings of both QD605 and QD655 are proprietary, it is likely that the differences in sensitivity are due to variations in this coating.

We have used two different analysis methods to identify directed motion, so our findings are unlikely to have arisen as a result of the analytical approach. However, a potential issue is that all trajectories were imaged in 2D, effectively projecting what is in reality 3D movement onto a 2D plane. Although neuronal processes grown in culture (without glia on the coverslip) represent an exceptionally flat system, we also minimized the impact of this by monitoring changes in the point spread function and excluding trajectories with greater than 15% change. Critically, however, projection from 3D to 2D results in a reduction in identified instances of directed motion (Dupont et al., 2013). We are therefore confident that directed motion is not over-represented in our system, and indeed may be under-represented.

Using electrical stimulation, we find that the directed motion of EphB2Rs is activity dependent, where increased activity results in receptors spending more time undergoing this form of motion, without changing overall speed. It is well known that activity can affect the diffusional movement of receptors in neuronal membranes (Choquet and Triller, 2013). Synaptic activation and elevations of intracellular calcium levels can regulate the diffusion of dendritic AMPARs (Borgdorff and Choquet, 2002, Heine et al., 2008), predominantly in the extrasynaptic space (Groc et al., 2004). Similarly, activity affects the lateral diffusion of GABA_A_ receptors in a calcium-dependent manner (Bannai et al., 2009, de Luca et al., 2017). Interestingly, the calcium-influx-dependent reduction in GABA_A_ receptor DC is also restricted to dendritic regions between synapses, and this may provide a mechanism for transfer of an “activation memory” between inhibitory synapses (de Luca et al., 2017). Recently, tracking of intracellular transport vesicles has demonstrated that increased neuronal activity (via “chemical long-term potentiation” [cLTP]) resulted in elevated anterograde velocities of AMPARs undergoing directed movement in vesicles, in the later stages of cLTP (Hangen et al., 2018). It is apparent that neurons require methods to alter the distribution of key receptors in response to changes in activity levels and patterns, and directed membrane flow could represent an additional mechanism.

The dominant model of cell membrane structure remains the fluid mosaic model (Goni, 2014, Singer and Nicolson, 1972) where both proteins and lipids move within the fluid lipid bilayer, which is often described as having the consistency of olive oil (Edidin, 2003). This fluidity includes both translational and rotational (about their own axis) movement of molecules. In biological membranes, cholesterol is thought to “buffer” fluidity, increasing fluidity at lower temperatures and reducing it at higher ones (Alberts, 2015, Maxfield and van Meer, 2010). Cholesterol is also associated with a liquid ordered phase where rotational movement is restricted (Goni, 2014), possibly in the context of lipid nanodomains, or “rafts” (Simons and Ikonen, 1997, Simons and van Meer, 1988). Indeed, high-resolution live cell imaging studies have indicated that cholesterol plays a role in “trapping” sphingolipids and glycosylphosphatidylinositol (GPI)-anchored proteins (Dietrich et al., 2002, Suzuki et al., 2012, Wenger et al., 2007). Consistent with this, our finding of significant increases in mean values of α for both super-confined and confined diffusing EphBRs, together with the overall increase in DC, suggests release from such domains. Although manipulation of cholesterol levels can also have effects on the actin cytoskeleton (Goodwin et al., 2005, Kwik et al., 2003, Sun et al., 2007), including the organization of GPI-anchored proteins in the membrane (Goswami et al., 2008), and indeed actin-dependent, directional membrane flows have recently been described (Ashdown et al., 2017), the lack of effect on directed motion from modulation of the actin network argues that this is not actin mediated, but rather dependent on the membrane itself. A possibility is that highly transient fluid currents may provide the conduits for directional movement of proteins in the neuronal membrane. In a polarized cell such as a neuron, with the need to rapidly move proteins in response to stimuli such as activity, this could represent an alternative and energy-efficient method of protein delivery.

### Limitations of the Study

This work was carried out *in vitro* using cultured rat neurons. Although it remains very challenging to perform SPT in mammalian neurons *in vivo*, especially where antibodies to specific proteins are used as here, it will be critical to devise new methods in the future to allow confirmation of findings in more intact preparations. In addition, the development of new, alternative probes, especially ones that can be genetically encoded and are smaller in size than those used here, will aid validation. We have discussed the limitations of imaging in 2D versus 3D in the Discussion section. Although we demonstrate the impact of cholesterol depletion on directed motion, it will be important to assess whether more subtle or different ways of changing or disrupting membrane lipids also affect this behavior.

## Methods

All methods can be found in the accompanying Transparent Methods supplemental file.

## Supplemental Information

Document S1. Transparent Methods and Figures S1–S3
