# Supplementary material for: Neuronal Receptors Display Cytoskeleton-Independent Directed Motion on the Plasma Membrane
Source: iScience. 2018 Dec 5;10:234–44. doi: 10.1016/j.isci.2018.12.001 (PMC6297241; doi:10.1016/j.isci.2018.12.001)
Supplement: Document S1. Transparent Methods and Figures S1–S3 [file mmc1.pdf]

ISCI, Volume *10*

Supplemental Information

Neuronal Receptors Display Cytoskeleton-Independent Directed Motion on the Plasma Membrane

Ruth D. Taylor, Martin Heine, Nigel J. Emptage, and Laura C. Andreae

Supplemental Figures

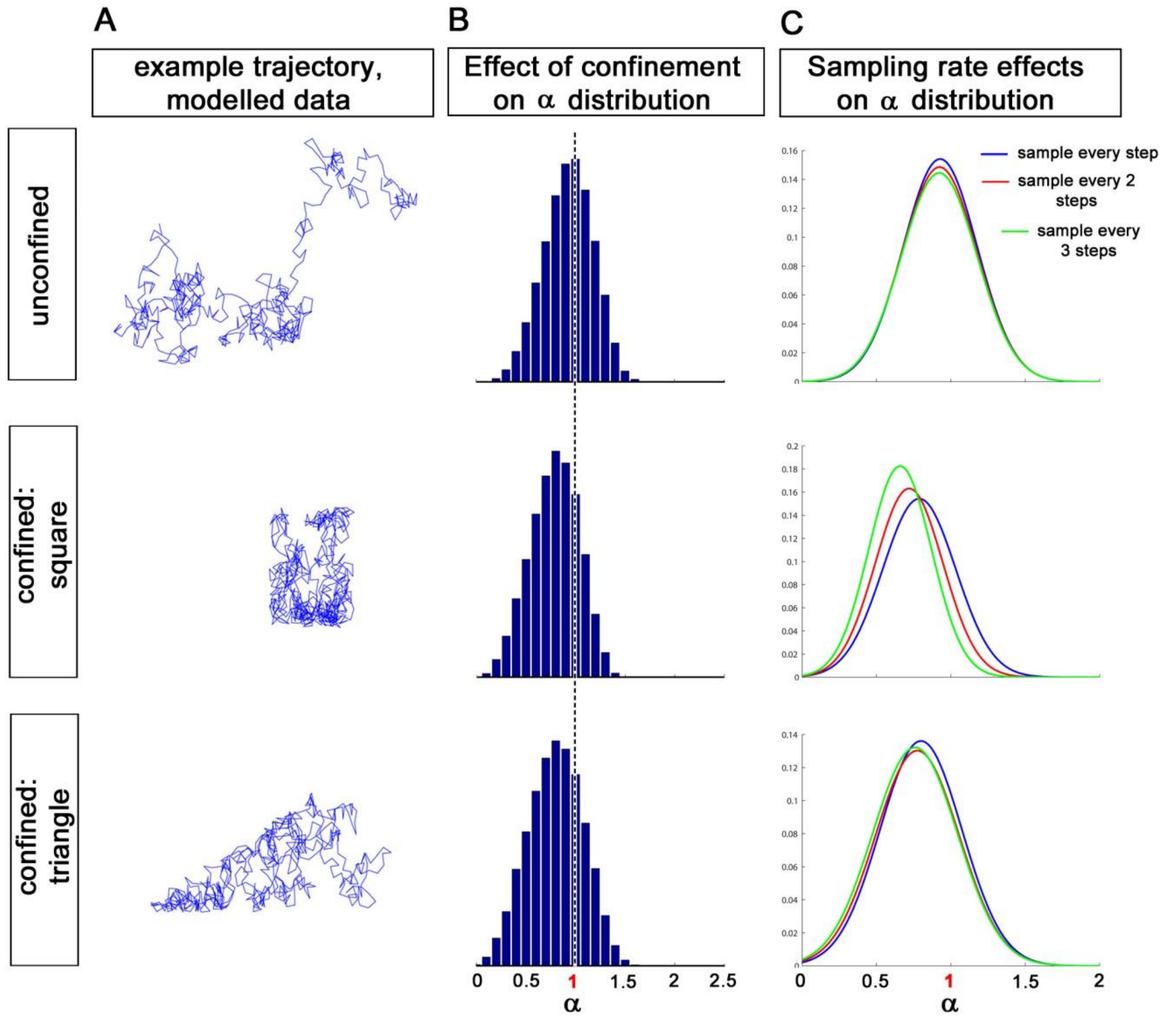

Figure S1. The effect of confinement geometry and sampling rate on motion characteristics (α) of diffusing particles. Related to Figure 2.

Particle movement modelled as diffusion, with time-resolved analysis. (**A**) Example trajectories of individual particles moving without confinement (top), within a confined square (middle) and a confined triangle where trajectory start point is in acute angle corner (bottom). (**B**) Distribution of α for each type of confinement shown in (**A**) indicates that as predicted, α peaks at 1 for unconfined movement and is shifted downwards where movement is confined. No variant in the geometry of confinement resulted in any examples of super-diffusive movement. (**C**) The effect of sampling rate on the distribution of α under different types of confinement, as shown in (**A**), indicates minimal effect on unconfined movement and a shift towards lower values of α with confinement.

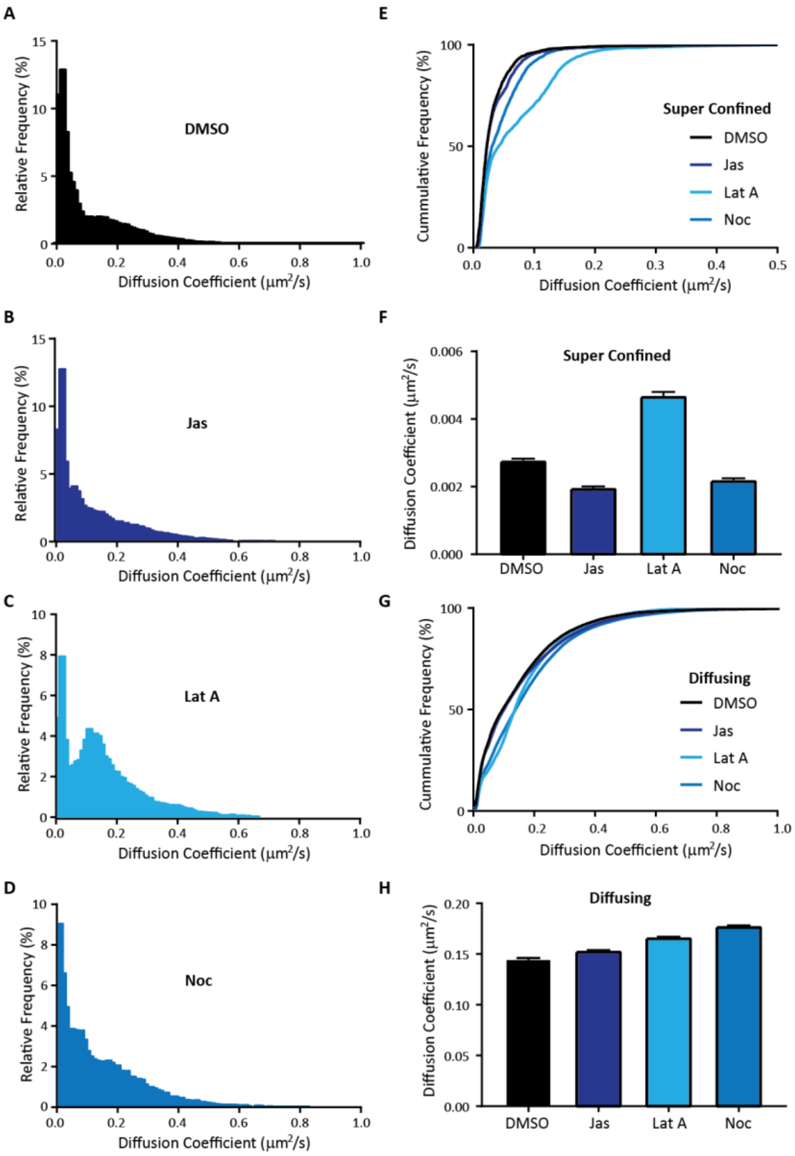

Figure S2. The effect of cytoskeletal modulatory drugs on diffusing QD-EphB2Rs. Related to Figure 3. **(A-D)** The distribution of instantaneous diffusion coefficients associated with diffusing QD-EphB2Rs in control (**A**), compared with modulating the actin cytoskeleton with 5 µM Jasplakinolide (**B**) and 5 µM Latrunculin A(**C**), or disrupting the microtubule network with 50 µM Nocodazole (**D**). The distributions were separated into two populations, super confined (**E**, **F**) and diffusing (**G**, **H**), based on a double gaussian fit to the distribution of α values that were calculated alongside the diffusion coefficient. An instantaneous diffusion coefficient was defined as belonging to a distribution if its corresponding α value was within the mean ± standard deviation for that distribution. (**E**, **G**) Cumulative frequency plots of the diffusion coefficients associated with the super confined (**E**) and diffusing (**G**) populations. (**F**, **H**) The mean of the distribution of diffusion coefficients ($\mu m^2/s$) for super confined (**F**; DMSO: 0.0337±0.0005; Jasplakinolide: 0.0366±0.0006; Latrunculin A: 0.0693±0.0012; Nocodazole: 0.0456±0.0008) and diffusing (**H**; DMSO: 0.1456±0.0004; Jasplakinolide: 0.1535±0.0004; Latrunculin A: 0.1665±0.0005; Nocodazole: 0.1778±0.0004) populations in the different cytoskeletal modulatory drugs. All distributions were significantly different from control (one way ANOVA; $p<0.0001$), error bars represent SEM.

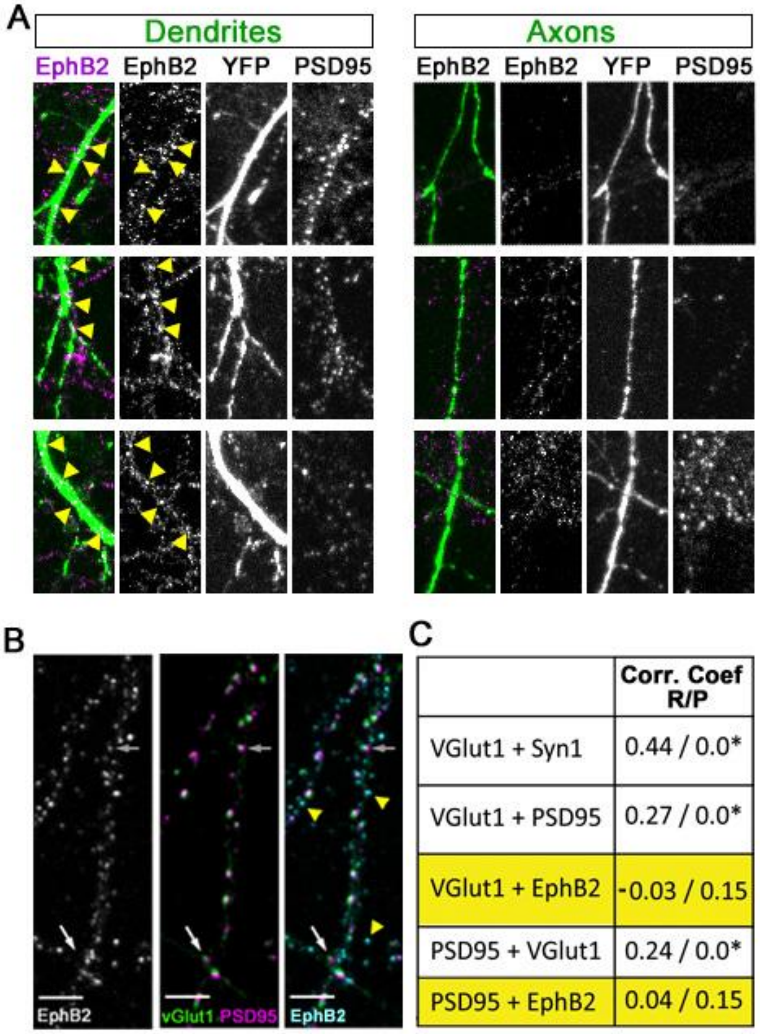

	Corr. Coef R/P
VGlut1 + Syn1	0.44 / 0.0*
VGlut1 + PSD95	0.27 / 0.0*
VGlut1 + EphB2	-0.03 / 0.15
PSD95 + VGlut1	0.24 / 0.0*
PSD95 + EphB2	0.04 / 0.15

Figure S3. EphB2 Receptor localization in cultured hippocampal neurons. Related to Figure 4.
(**A**) Examples of YFP-expressing dendrites (left panels) and axons (right panels) co-stained with EphB2 and PSD95 indicates that EphB2 (magenta) is predominantly found on dendrites (yellow arrowheads).
(**B**) Excitatory synapses, labelled with the presynaptic marker vGlut1 (green) and postsynaptic marker PSD95 (magenta), are both EphB2 positive (grey arrow) and negative (white arrow), but the majority of EphB2 puncta (cyan) are extrasynaptic (yellow arrowheads). Scale bars 5 µm. Quantification shown in (**C**): automated puncta analysis (custom written MATLAB routines) identified labelled puncta and colocalization assessed by calculating correlation coefficients (MATLAB). As a positive control, colocalization between two known presynaptic markers, VGlut1 and synapsin1 (Syn1), showed a correlation coefficient of 0.44 ($p < 0.001$). Also significant ($p < 0.001$) with a less strong correlation was that between an excitatory presynaptic marker, vGlut1 and a postsynaptic marker, PSD95, r = 0.27, or r = 0.24 for PSD95 vs vGlut1, which should tightly overlap. In contrast, there was no correlation seen between EphB2 and vGlut1 (r = -0.03) nor between EphB2 and PSD95 (r = 0.04), indicating that EphB2 is predominantly not localized at or close to synapses.

Transparent Methods

Hippocampal Cultures and Transfection

Dissociated hippocampal neuronal cultures were prepared from embryonic day 18 Sprague Dawley rats of both sexes in accordance with all institutional and national guidelines. Hippocampi were dissociated using trypsin (5 mg/ml for 15 min at 37°C; Worthington), triturated through narrow diameter Pasteur pipettes and plated at 350 cells/mm^2 on glass coverslips pre-coated with poly-d-lysine (50 µg/ml; Sigma) and laminin (20 µg/ml). Neurons were incubated (37°C, 6% CO_2) in a 50:50 mixture of neurobasal media supplemented with B27 (2%) and neurobasal media with fetal calf serum (2%), with additional glutamax (500 µM) and penicillin-streptomycin (100 µg/ml). At 7 days *in vitro* (DIV) half of this medium was replaced with neurobasal medium plus B27 supplement. Unless otherwise specified, all culture reagents were from Gibco.

Neurons (9-10 DIV) were transfected with PSD-95-GFP or YFP (Synapsin promoter) using Effectene Transfection Reagent (QIAGEN) according to the manufacturer's instructions.

Live Single Molecule Optical Microscopy

Dissociated hippocampal neurons (10-14 DIV) were incubated for 10 minutes at 37°C in conditioned cell culture medium containing 1% casein and 1% pre-coupled QD-EphB2R mix. Pre-coupling was carried out by incubating QDot 655 (rabbit F(ab')2-anti goat IgG (H+L) conjugate (H+L); Cat no: Q11821MP, Fisher) with EphB2R antibody (BD Pharmingen) in a 1:4 ratio in sterile PBS for 1 hour at room temperature (RT). Neurons were subsequently washed and imaged in Tyrode's solution. Images were acquired <20 minutes after the staining protocol was complete.

Tyrode's solution contained: 128 mM NaCl, 5 mM KCl, 1 mM $MgCl_2$, 2 mM $CaCl_2$, 15 mM HEPES, 4 mM $NaHCO_3$, and pH was adjusted as required with HCl. NH_4Cl was used to dissipate pH gradients as previously described (Andreae et al., 2012). Temperature was maintained at 35°C for the duration of the experiments with the aid of an objective warmer and heated chamber (Harvard Apparatus). Images were acquired using an Olympus IX71 inverted microscope with a 100X, 1.4 NA oil immersion objective, coupled to a Photometrics Evolve EMCCD camera and associated Slidebook software (Intelligent Imaging Innovations, Denver). Illumination was provided by an LED emitting at 470 nm. The filter cube used for QD imaging contained a dichroic mirror at 475 nm and a 40 nm emission filter centred at 655 nm. Filters were purchased from Chroma Technologies. QD imaging movies were acquired with an acquisition rate of 32.9 Hz and were 3000 frames in length.

Electrical stimulation experiments were conducted using a field stimulation chamber (Harvard Apparatus) coupled to a SD9 stimulator delivering 900 pulses at 20 Hz. Changes in the motion of QD-EphB2Rs were analysed 5 minutes after delivery of electrical stimulation.

Filipin Fluorescence to measure Cholesterol Depletion

After treatment with methyl-β-cyclo-dextrin (1 mM, 30 mins at 37°C), dissociated hippocampal neurons (10-14 DIV) were fixed in 4% paraformaldehyde for 10 minutes and permeabilized with 0.01% saponin . Cells were then incubated with filipin (Sigma) complex (100 μg/ml) for five minutes and imaged immediately to avoid photo-bleaching.

Filipin fluorescence was measured using the ImageJ plugin: NeuronJ to trace neuronal processes, and then a custom written MATLAB (Mathworks) routine to measure the fluorescence intensity of the traced processes and subtract background from the images.

Immunostaining

Immunocytochemistry against EphB2Rs was conducted prior to cell fixation. Dissociated hippocampal neurons were blocked with casein (10%, 10 min, 37°C) and then subsequently incubated with primary antibody to EphB2R (1:500, 30 min, 37°C) and secondary antibody (1:500, 10 min, 37°C). Cells then underwent methanol fixation (100%, 1 min, -20°C) and were permeabilized (0.1% saponin, 10 min) and blocked (3% BSA, 30 min) prior to incubation with primary (1 hour at RT) and secondary antibodies (30 min at RT). The primary antibodies used were: mouse anti-PSD95 (Pierce), rabbit anti-vGLUT1 (Synaptic Systems), chick anti-GFP (Abcam).

Drugs and Reagents

Unless otherwise specified, all drugs were added to the recording chamber for 10 minutes prior to recording, dissolved in DMSO (final concentration 0.05%) and purchased from Sigma-Aldrich: methyl-β-cyclo-dextrin, filipin complex, jasplakinolide (Tocris), latrunculin A (Tocris), nocodazole and QSY-21 (Life Technologies). For experiments with low dose nocodazole (200 nM) cells were incubated in the drug for 4 hours are 37°C prior to recording.

Pre-clustered ephrinB1-Fc was obtained by incubating ephrinB1-Fc (R&D Systems) with antibodies against Fc fragments (Sigma) in a 1:10 ratio for 1hour at 37°C. The mixture was then maintained until use at 0°C. Pre-clustered ephrinB1-Fc was applied to the cells at a final concentration of 2.2 μg/ml. In control experiments Fc fragments replaced ephrinB1-Fc at an equivalent concentration.

Receptor Tracking

Initially, the shape of the point spread function (PSF) of individual QD-EphB2Rs was analysed. The change in the diameter of the PSF of individual QD-EphB2Rs over the course of an experiment was measured using the Fiji plugin Trackmate (Tinevez et al., 2017). In order to calibrate the QD-EphB2R motion in the Z plane, immobilised QDs were imaged with 100 nm step changes in focus. Individual QD-EphB2Rs where the PSF changed by more than 15% in diameter were excluded and not analysed further, this equated to <0.5 μm shift in the Z direction. The imaged molecules were detected and tracked in MATLAB using the plugin software UTrack 2.1 (Jaqaman et al., 2011). The individual QD-EphB2R tracks were then further analysed in MATLAB using custom written algorithms previously described (Arcizet et al., 2008).

Briefly, the displacement between every pair of sub-resolution co-ordinates was analysed enabling the mean squared displacement (MSD) to be calculated and the trajectory motion mode to be assigned. We calculate time resolved MSD by calculating the local MSD function over 40 frames. The local MSD function is calculated according to:

$$\Delta R2t(\delta t) = \langle(\boldsymbol{R}(t' + \delta t) - \boldsymbol{R}(t'))2\rangle - (T/2) < t' < (T/2)$$

The diffusion coefficient (D) was calculated by a linear least squares regression fit of the first three points of this function.

The motion mode is determined by fitting the power law and calculating α over the first twenty points of the MSD function:

$$\Delta R2t(\delta t) = A\delta t^{\alpha}$$

QD-EphB2Rs were determined to have a velocity when two criteria were satisfied: α greater than the critical value (1.561) and the statistical assessment of a polynomial fit to the MSD function was significantly better than a linear fit. The critical value for α was determined as being significantly greater than the α calculated for simulated random motion and is in agreement with Caspi and colleagues where a value of α>1.5 is synonymous with super diffusion (Caspi et al., 2000). Instantaneous velocity (V) was calculated for segments of a trajectory where α> 1.561 for at least two time steps.

The time resolved MSD was calculated using sliding windows of 40 steps to enable extrapolation of instantaneous motion characteristics. This enabled automatic trajectory analysis of motion modes without *a priori* segmentation.

To compare experimental data with simulated, we used trajectories without blinks and simulated 350 trajectories of similar lengths displaying random motion. The simulated trajectories were then analysed using time resolved MSD analysis and the distribution of instantaneous α obtained. The values of instantaneous α obtained for random simulated trajectories was 0.928±0.248.

Divide and conquer moment scaling spectrum (DC-MSS) analysis was conducted in MATLAB using the plugin software DC-MSS as described (Vega et al., 2018).

Data and Statistics

Unless stated otherwise in the text, all statistics are presented as the mean ± standard deviation. Statistical analysis was carried out using the Student t test, ANOVA or Chi-squared test where appropriate.

Data availability

https://data.mendeley.com/datasets/rwby5j793s/draft?a=1b88bb10-39ba-45e6-81f1-84306a39e0b4

Supplemental References

Andreae, L.C., Fredj, N.B., and Burrone, J. (2012). Independent vesicle pools underlie different modes of release during neuronal development. The Journal of neuroscience : the official journal of the Society for Neuroscience *32*, 1867-1874.

Arcizet, D., Meier, B., Sackmann, E., Radler, J.O., and Heinrich, D. (2008). Temporal analysis of active and passive transport in living cells. Physical review letters *101*, 248103.

Caspi, A., Granek, R., and Elbaum, M. (2000). Enhanced diffusion in active intracellular transport. Physical review letters *85*, 5655-5658.

Jaqaman, K., Kuwata, H., Touret, N., Collins, R., Trimble, W.S., Danuser, G., and Grinstein, S. (2011). Cytoskeletal control of CD36 diffusion promotes its receptor and signaling function. Cell *146*, 593-606.

Tinevez, J.Y., Perry, N., Schindelin, J., Hoopes, G.M., Reynolds, G.D., Laplantine, E., Bednarek, S.Y., Shorte, S.L., and Eliceiri, K.W. (2017). TrackMate: An open and extensible platform for single-particle tracking. Methods *115*, 80-90.

Vega, A.R., Freeman, S.A., Grinstein, S., and Jaqaman, K. (2018). Multistep Track Segmentation and Motion Classification for Transient Mobility Analysis. Biophysical journal *114*, 1018-1025.
